# *PbGA2ox8* induces vascular-related anthocyanin accumulation and contributes to red stripe formation on pear fruit

**DOI:** 10.1038/s41438-019-0220-9

**Published:** 2019-12-01

**Authors:** Rui Zhai, Zhigang Wang, Chengquan Yang, Kui Lin-Wang, Richard Espley, Jianlong Liu, Xieyu Li, Zhongying Wu, Pengmin Li, Qingmei Guan, Fengwang Ma, Lingfei Xu

**Affiliations:** 1 College of Horticulture, Northwest A&F University, Taicheng Road No. 3, Yangling, Shaanxi Province China; 2 The New Zealand Institute for Plant & Food Research, Ltd., (Plant and Food Research), Mt. Albert Research Centre, Private Bag, 92169 Auckland, New Zealand; 3 Horticultural Research Institute, Henan Academy of Agricultural Sciences, Huayuan Road No. 116, Zhengzhou, Henan Province China; 4 State Key Laboratory of Crop Stress Biology for Arid Areas, Northwest A&F University, Taicheng Road No. 3, Yangling, Shaanxi Province China

**Keywords:** Gibberellins, Secondary metabolism

## Abstract

Fruit with stripes, which are generally longitudinal, can occur naturally, but the bioprocesses underlying this phenomenon are unclear. Previously, we observed an atypical anthocyanin distribution that caused red-striped fruit on the spontaneous pear bud sport “Red Zaosu” (*Pyrus bretschneideri* Rehd.). In this study, comparative transcriptome analysis of the sport and wild-type “Zaosu” revealed that this atypical anthocyanin accumulation was tightly correlated with abnormal overexpression of the gene-encoding gibberellin (GA) 2-beta-dioxygenase 8, *PbGA2ox8*. Consistently, decreased methylation was also observed in the promoter region of *PbGA2ox8* from “Red Zaosu” compared with “Zaosu”. Moreover, the GA levels in “Red Zaosu” seedlings were lower than those in “Zaosu” seedlings, and the application of exogenous GA_4_ reduced abnormal anthocyanin accumulation in “Red Zaosu”. Transient overexpression of *PbGA2ox8* reduced the GA_4_ level and caused anthocyanin accumulation in pear fruit skin. Moreover, the presence of red stripes indicated anthocyanin accumulation in the hypanthial epidermal layer near vascular branches (VBs) in “Red Zaosu”. Transient overexpression of *PbGA2ox8* resulting from vacuum infiltration induced anthocyanin accumulation preferentially in calcium-enriched areas near the vascular bundles in pear leaves. We propose a fruit-striping mechanism, in which the abnormal overexpression of *PbGA2ox8* in “Red Zaosu” induces the formation of a longitudinal array of anthocyanin stripes near vascular bundles in fruit.

## Introduction

Fruit with stripes, which generally run longitudinally, can occur naturally. Alternating dark and light-green stripes are common in watermelon, pumpkin, and squash. Although such stripes form along the main carpellary vascular bundles^1,2^, the molecular mechanism of stripe formation has not been investigated. Unlike the common green stripes in watermelon, red stripes sometimes occur in apple, pear, and peach. The red appearance of these fruits results from anthocyanin accumulation^3–5^. Nevertheless, the genetic basis of this anthocyanin-associated striping trait is poorly understood, as are the mechanisms underlying the formation and distribution of the stripes.

Anthocyanins not only accumulate in flowers to attract insects for pollen dispersal or in fruit to attract animals for seed dispersal but also are produced in plants as radical scavengers in response to various biotic and abiotic stresses^6,7^. The signaling networks behind the developmental and environmental regulation of anthocyanin biosynthesis are complex. Light stimuli are necessary for the production of anthocyanins in most plants. The ubiquitin E3 ligase CONSTITUTIVE PHOTOMORPHOGENIC1, ELONGATED HYPOCOTYL5, and PHYTOCHROME-INTERACTING FACTOR 3 are key components involved in the regulation of light-dependent anthocyanin biosynthesis^8^. The activities of ELONGATED HYPOCOTYL5 and PHYTOCHROME-INTERACTING FACTOR 3 can be modulated by DELLA proteins involved in gibberellin (GA) signaling^9,10^. The regulation of anthocyanin accumulation induced by different kinds of internal or external stimuli, such as jasmonate, temperature, sucrose, and nutritional starvation^10–12^, is affected by GA–DELLA signaling. Moreover, calcium signals may participate in anthocyanin biosynthetic regulatory networks that are induced by abscisic acid, sucrose, or environmental stimuli, including drought or low temperature^13–15^.

Anthocyanins, such as cyanidin 3-galactoside in pear, are derived from phenylalanine, the common precursor of flavonoid metabolites, through a series of enzymatic actions^16^. Among the flavonoid biosynthetic enzymes, dihydroflavonol 4-reductase, UDP-glucose: flavonoid 3-glucosyltransferase (UFGT), and anthocyanidin synthase are directly involved in anthocyanin biosynthesis^16^. The transcript levels of the genes encoding these anthocyanin biosynthetic enzymes are directly regulated by the MYB–bHLH–WD40 transcription complex^17^. Several MYB transcription factors, including *PbMYB10*, *PbMYB10b* (*PbMYB114*), and *PbMYB9*, are involved in pear anthocyanin regulation^18,19^.

**Fig. 1:**
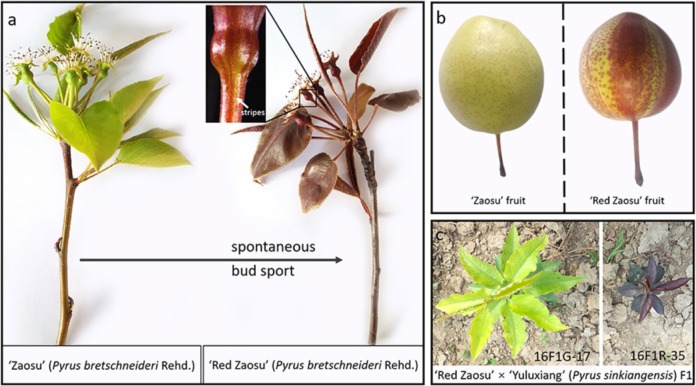
Appearance of the “Zaosu” pear, “Red Zaosu” sport and F1 hybrids from a “Red Zaosu” × “Yuluxiang” cross. **a** Young leaves and receptacles of “Zaosu” and “Red Zaosu”. **b** Mature fruit of “Zaosu” and “Red Zaosu”. **c** A green F1 plant (labeled 16F1G-17) and a red F1 plant (labeled 16F1R-35) among the progeny of a “Red Zaosu” × “Yuluxiang” cross.

Previously, we reported “Red Zaosu” as a spontaneous bud sport of the “Zaosu” common Chinese pear^20^. Anthocyanins accumulated mostly in leaf-derived tissues and produced red leaves and red-striped fruit skins in “Red Zaosu” (Fig. 1a, b). We found red:green plant segregation ratios of 102:116 and 48:51 in two F1 populations derived from a “Red Zaosu” × “Yuluxiang” cross in 2015 (15F1) and 2016 (16F1), respectively (Fig. 1c). Because of this finding, we proposed that the red trait (R) is controlled by a single dominant gene.

In this study, pear GA 2-beta-dioxygenase 8 (*PbGA2ox8*) was confirmed to contribute to this atypical anthocyanin accumulation pattern in “Red Zaosu”. We observed that calcium-enriched xylem cells were spatially coordinated with anthocyanin distribution near vascular bundles, and this coordination was necessary for anthocyanin production in the fruit skin of the sport. *PbGA2ox8*-related anthocyanin accumulation was demonstrated to be sensitive to these calcium-enriched areas and resulted in the formation of anthocyanin-associated stripes near vascular bundles in pear fruit.

## Materials and methods

### Material preparation and explant propagation

“Red Zaosu”, a spontaneous bud sport of “Zaosu”, was first found in a commercial orchard in Linwei District, Weinan City, Shaanxi Province, China, in 2004. In this study, “Red Zaosu” (*P. bretschneideri* Rehd.), “Zaosu” *(P. bretschneideri* Rehd.), “Red Anjou” (*Pyrus communis* L.), “Hong sichou” (*P. communis* L.), “Early Red Comice” (*P. communis* L.), “Dangshansu” (*P. bretschneideri* Rehd.), “Bartlett” (*P. communis* L.), and “Suisho” (*Pyrus pyrifolia* Nakai.) pear were collected from the horticultural research base of Northwest A&F University in Yangling District, Shaanxi Province, China. F1 plants from a “Red Zaosu” × “Yuluxiang” (*Pyrus sinkiangensis* Yu) cross were collected from the horticultural research base of the Henan Academy of Agricultural Sciences in Xinxiang County, Henan Province, China. “Red Zaosu” and “Zaosu” explants were propagated at the Laboratory of Fruit Trees Stress Biology of the College of Horticulture, Northwest A&F University. The detailed culture conditions, tissue information, information on the application of GA, and the calcium channel blocker lanthanum chloride (LaCl_3_) and sampling dates for these materials are listed in Supplementary Table S1. Fresh plant tissues were immediately frozen, powdered in liquid nitrogen, and stored at −80 °C for later use.

### DNA and RNA extraction and purification

The total DNA and RNA were both extracted and purified using SDS solubilization and phenol extraction, respectively^21^.

### RNA sequencing and analysis

The total RNA (3 μg) extracted from the receptacles, young leaves, and mature leaves of “Red Zaosu” and “Zaosu” and red/green-striped regions of “Red Zaosu” fruit were used for sequencing, with three biological replicates for each material assessed. Briefly, the total RNA was randomly fragmented, reverse-transcribed, amplified, and purified to form a cDNA library. After the cDNA library was assessed using the Bioanalyzer 2100 system (Agilent, CA, USA), the library preparations were paired-end sequenced (100 bp) on a HiSeq 2500 platform (Illumina, CA, USA). Clean reads were enriched by removing reads containing adapters, reads with multiple unknown bases, and low-quality reads from the raw data. Paired-end clean reads were aligned to the pear genome using TopHat^22^. Genes with *q*-values < 0.05 as assessed by the DESeq R package and fold changes in expression >2 were assigned as differentially expressed genes (DEGs)^23^. The functional annotations and gene ontology (GO) terms enriched in most DEGs were characterized using the pear genome as a reference. The annotations and GO terms enriched in 26 DEGs common to young leaves, mature leaves, and receptacles were further adjusted using any previously published orthologs. Variations in the expression levels and GO terms enriched in the 26 DEGSs were visualized using the GOChord R package^24^. The DEGs between red/green-striped regions of “Red Zaosu” fruit were clustered by the KEGG pathway enrichment analysis using DAVID^25^.

### Quantitative real-time PCR

Purified RNA (2 μg) was reverse-transcribed to cDNA using the PrimeScript RT reagent kit with gDNA Eraser (TaKaRa, Dalian, China). The primer pairs for selected genes and *PbActin* (an internal control) are listed in Supplementary Table S2. PCR was performed on a StepOnePlus PCR system (ABI, USA) with SYBR Premix Ex Taq II (TaKaRa, Dalian, China) according to the manufacturer’s instructions. Expression data from three biological replicates were analyzed using the cycle threshold (2^−ΔΔCt^) method.

### Histological analysis

Fresh latitudinal sections of the receptacles of “Red Zaosu” and “Zaosu” were manually cut and temporarily preserved in a 5% (w/v) ascorbic acid solution during microscopic observation. Paraffin sectioning of the “Red Zaosu” receptacle was performed^26,27^. Fresh “Red Zaosu” flowers were immediately fixed in a formaldehyde–acetic acid–alcohol solution. The receptacles were separated, dehydrated, embedded in paraffin, cut into 10-μm slices, and stained with Fast green and safranin. The safranin-labeled xylem was imaged using a 450-nm excitation filter in combination with a 520–550-nm emission filter. Histological analysis was carried out using a BX51 + PD72 + IX71 microscopic imaging system (Olympus, Tokyo).

### Anthocyanin quantification

Anthocyanin extraction and quantification were performed as previously described^28^. Briefly, the extraction buffer was 70% methanol containing 2% formic acid. Extracted anthocyanin was filtered through a 0.45-μm syringe filter prior to HPLC analysis. The anthocyanin concentration was determined by the absorbance at 520 nm on an HP 1200 liquid chromatograph equipped with a diode array detector (Agilent, CA, USA). The anthocyanin in three biological replicates was quantified based on the calibration curve for a cyanidin 3-galactoside standard (Sigma-Aldrich, MO, USA).

### McrBC-PCR analysis

The DNA methylation level was analyzed using McrBC-PCR. In total, 1 μg of genomic DNA was isolated from the young leaves of “Zaosu”, “Red Zaosu”, and the 15F1 hybrid population. The isolated DNA was then digested with 40 units of the methylation-sensitive restriction enzyme McrBC (New England Biolabs; M0272L) for 2 h, and the digestion buffer without GTPase was used as the negative control. The methylation level in the digested DNA templates was measured by semiquantitative PCR. The 1300-bp region upstream of the ATG translation site of *PbGA2ox8* was divided into four overlapping fragments and amplified using McrBc-PCR. The primer pairs used are listed in Supplementary Table S2.

### Transient assay

The transient assay was performed as previously described, with a modified infiltration method^18^. The full-length *PbGA2ox8* complete coding sequence was PCR-amplified from “Red Zaosu” cDNA and then cloned into the pCambia1301 binary vector (replacing the GFP-coding sequence) using a ClonExpress One Step Cloning Kit (Vazyme) with homologous recombination technology. The primer pairs for selected genes and *PbActin* (an internal control) are listed in Supplementary Table S2. The plasmid was transferred into *Agrobacterium tumefaciens* strain GV3101 and suspended at 28 °C in the LB medium containing 10 mM MES and 20 μM acetosyringone with the appropriate antibiotics. *Agrobacterium* cells were harvested, resuspended in infiltration buffer (10 mM MgCl_2_, 10 mM MES, pH 5.6, and 100 μM acetosyringone) to a final OD_600_ of 0.8 and cultured for 4 h at room temperature before infiltration. Plant infiltration was performed by vacuum instead of injection. Young expanded leaves from cultured “Zaosu” explants were completely immersed and vacuumed in 2-mL *Agrobacterium* suspensions (containing pCambia1301-GA2ox8 and pCambia1301-GFP) at 25 kPa for 5 min. The GFP signals in leaves infiltrated with pCambia1301-GFP were imaged using a BX51 + PD72 + IX71 microscopic imaging system (Olympus). Infiltrated leaves were then cultured on MS medium containing different combinations of 0.1 M sucrose and 1 mM LaCl_3_ (the calcium channel blocker) for 5 days at 1200 lx.

### GA quantification

GA_4_ extracted from mature fruit skin and “Red Zaosu” and “Zaosu” explants was quantified as previously described^29,30^. Three biological replicates were assessed. Samples of 200 mg were ground in 1.5 mL of extraction buffer (20% methanol, 79% isopropanol, and 1% acetic acid) at 4 °C. The supernatant was filtered through a 0.22-μm syringe filter prior to HPLC analysis. LC-MS^2^ analysis was performed using an HPLC system (Agilent 1290) coupled to a SCIEX 6500 Qtrap (AB Sciex). Samples were injected onto ZORBAX SB-C18 columns (2.1 × 150 mm, 3.5 μm; Agilent). GA_4_ was screened and quantified using the multiple reaction monitoring model with a transition of 331.0 > 213.0.

### Calcium imaging and calcium concentration quantification

The artificially shaded young leaves of “Red Zaosu” explants, artificially bagged carpopodia of “Red Zaosu”, naturally growing young leaves of “Zaosu” explants, and naturally growing carpopodia of “Zaosu” were incubated with fluorescence dye (40 μM Fluo-3/AM ester, 0.2 mM CaCl_2_, and 50 mM sorbitol) at 4 °C for 5 min in the dark. The calcium signal was assessed using a 450-nm excitation filter in combination with a 520–550-nm emission filter on a BX51 + PD72 + IX71 microscopic imaging system (Olympus). The total calcium concentration was quantified by flame atomic absorption spectrophotometry.

### Statistical analysis

To test the significance of differences among the data, one-way analysis of variance with Tukey’s honestly significant difference test was conducted using SPSS 16.0 software.

### Accession numbers

Sequence information for the genes used in this study is listed in Supplementary Table S2.

## Results

### Atypical overexpression of *PbGA2ox8* was tightly correlated with anthocyanin accumulation in the “Red Zaosu” sport

To investigate the molecular mechanisms underlying anthocyanin accumulation and distribution in “Red Zaosu”, mRNA sequence variations and DEGs between the leaves and fruitlets of “Red Zaosu” and “Zaosu” were screened using comparative transcriptome analysis. “Red Zaosu” is a very young sport that mutated from “Zaosu” <20 years ago. As expected, in this study, no valuable SNPs or InDel sites associated with the red trait were identified in this sport.

**Fig. 2:**
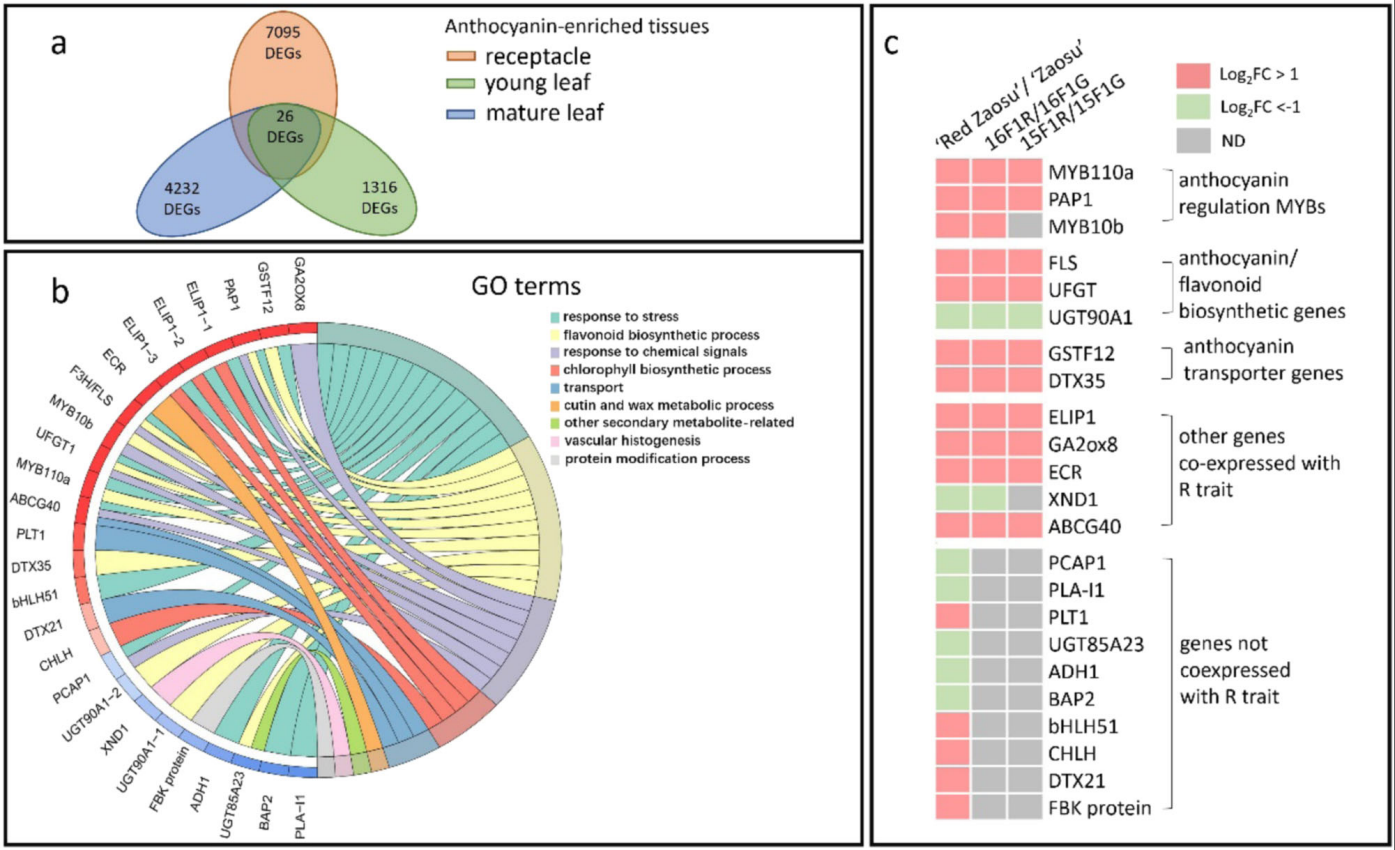
Overlapping differentially expressed genes (DEGs) from different anthocyanin-enriched tissues between “Red Zaosu” and “Zaosu”. **a** A Venn diagram showing the number of overlapping DEGs between “Red Zaosu” and “Zaosu” from the receptacle, young leaf, and mature leaf. **b** Gene ontology (GO) classification of these 26 DEGs. The fold changes (FCs) of the DEGs were normalized and are presented as Log_2_FC-Red/Green values. **c** Identification of genes coexpressed with the red trait of “Red Zaosu”. The FCs in expression of the 26 DEGs between “Red Zaosu” and “Zaosu” and the pooled red/green-leafed segregants of the “Red Zaosu” × “Yuluxiangli” cross were normalized as Log_2_FC values and are presented as differentially colored rectangles using color to indicate changes in expression in Excel 2016. The gray-labeled rectangles represent FCs in expression that were less than twofold or represent a nonsignificant difference (ND). The expression levels of the 26 DEGs were quantified by qPCR. Three biological replicates were assessed, and the statistical significance of the differences was determined using Student’s *t* test in (**c**).

In total, 26 overlapping DEGs were screened from the anthocyanin-enriched tissues (fruitlets, young leaves, and mature leaves) between “Red Zaosu” and “Zaosu” (Fig. 2a; Supplementary Datasets S1, S2). The 26 DEGs were further annotated and classified into different GO terms. More than 50% of these DEGs were classified as environmental stress-response genes (Fig. 2b). This result indicated that even though “Red Zaosu” was grown under the same environmental conditions as “Zaosu”, the former was experiencing more stress.

Moreover, a relatively complete anthocyanin biosynthetic gene network was identified among the 26 DEGs, which included the anthocyanin regulatory transcription factor *PRODUCTION OF ANTHOCYANIN PIGMENT* (*PAP*) type *MYB*s (*PAP1*, *PAP2*, and *MYB110a*), the flavonoid biosynthetic genes *flavonol synthase* (*FLS*) and *UFGT*s, and the anthocyanin transporters *GLUTATHIONE S-TRANSFERASE F12* (*GSTF12*) and *DETOXIFICATION 35* (*DTX35*) (Fig. 2b).

To determine whether the 26 DEGs were associated with the red trait in “Red Zaosu”, their expression patterns were further investigated in pooled red/green-leafed segregants of “Red Zaosu” × “Yuluxiangli” crosses performed in 2015 (15F1R and 15F1G) and 2016 (16F1R and 16F1G). In total, 13 of the 26 DEGs, including all of the anthocyanin genes, were coexpressed with the red trait in “Red Zaosu” (Fig. 2c).

**Fig. 3:**
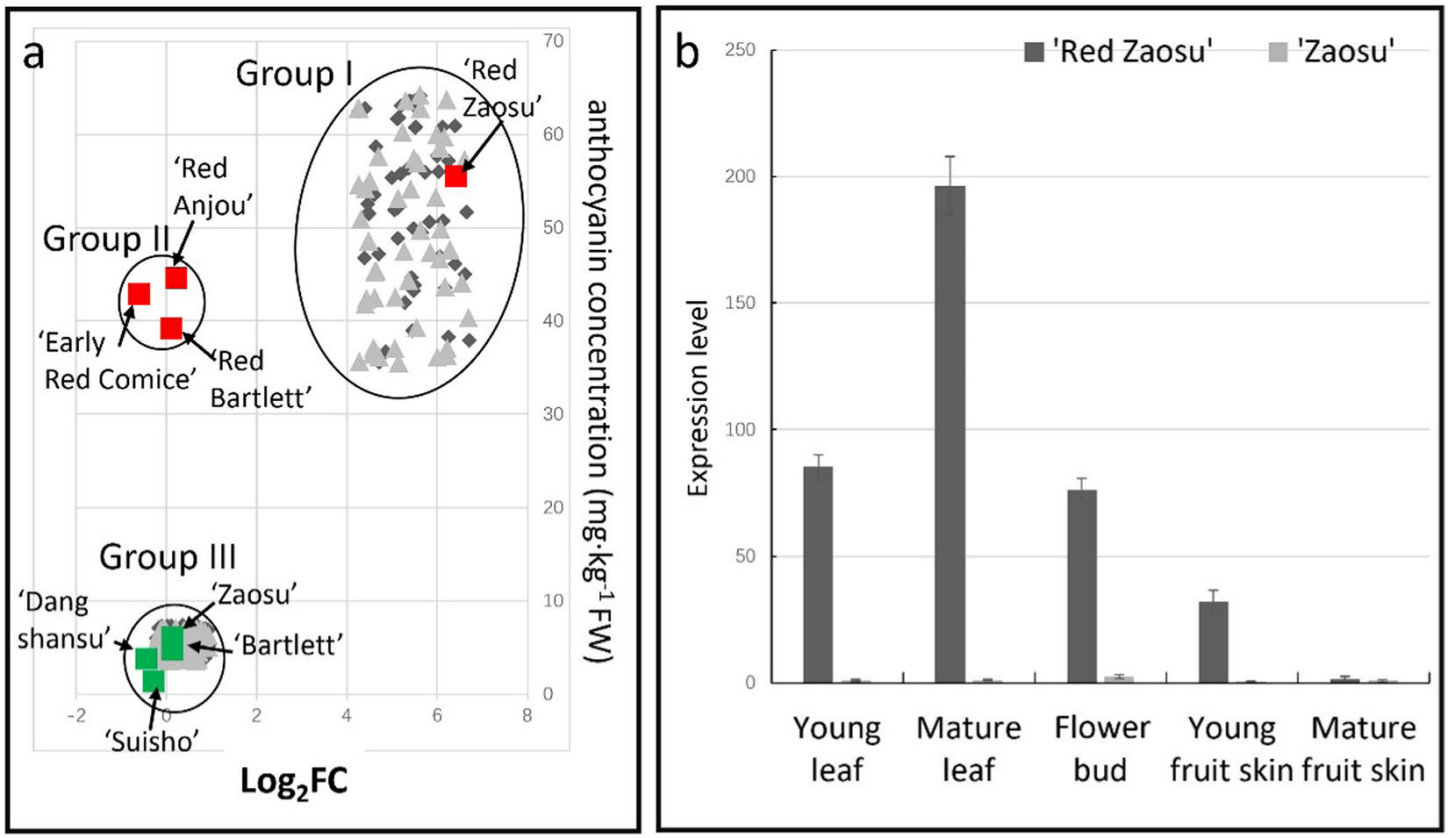
*PbGA2ox8* expression patterns. **a** The *PbGA2ox8* expression patterns in different pear cultivars as well as the 15F1 and 16F1 segregants. **b** The *PbGA2ox8* expression patterns in different tissues of “Red Zaosu” and wild-type “Zaosu”. The fold change (FC) in expression of *PbGA2ox8* was normalized and is presented as the Log_2_FC in (**a**). The 15F1 and 16F1 populations in (**a**) comprised 100 and 99 individuals, respectively. Error bars in (**b**) represent the means ± SEs of three biological replicates.

Among the 13 DEGs, the expression pattern of *PbGA2ox8* was unique in “Red Zaosu” (Fig. 3). Therefore, its expression was investigated in different pear cultivars, the 15F1 and 16F1 segregants, and different organs of “Red Zaosu” (Fig. 3). Based on the correlation between anthocyanin concentration and the expression pattern of *PbGA2ox8*, three different groups were identified. Group 1, which was characterized by the high expression of *PbGA2ox8*, consisted of “Red Zaosu”, 45 anthocyanin-enriched 15F1 lines, and 48 anthocyanin-enriched 16F1 lines. Other red pear cultivars (with red fruit but no stripes) were clustered into group II, in which the anthocyanin accumulation was high but *PbGA2ox8* was expressed at a low level. No progeny from the 15F1 or 16F1 cross was found in group II. Group III members, which had low anthocyanin concentrations and expressed low levels of *PbGA2ox8*, consisted of 55 anthocyanin-deficient 15F1 lines, 51 anthocyanin-deficient 16F1 lines, and the green pear cultivars, including “Zaosu” (Fig. 3a). This result indicated that *PbGA2ox8* was highly expressed exclusively in anthocyanin-enriched individuals in the 15F1 and 16F1 populations. Thus, the complete correlation between *PbGA2ox8* expression and anthocyanin accumulation was verified. Moreover, *PbGA2ox8* exhibited exclusive expression patterns in the anthocyanin-enriched organs of “Red Zaosu” (Fig. 3b). Thus, *PbGA2ox8* was expressed exclusively in the anthocyanin-enriched organs of “Red Zaosu”, but not in “Zaosu” or any other pear cultivars, even those with red fruit (Fig. 3).

### The promoter region of *PbGA2ox8* was demethylated exclusively in “Red Zaosu” and its red-leafed progeny

**Fig. 4:**
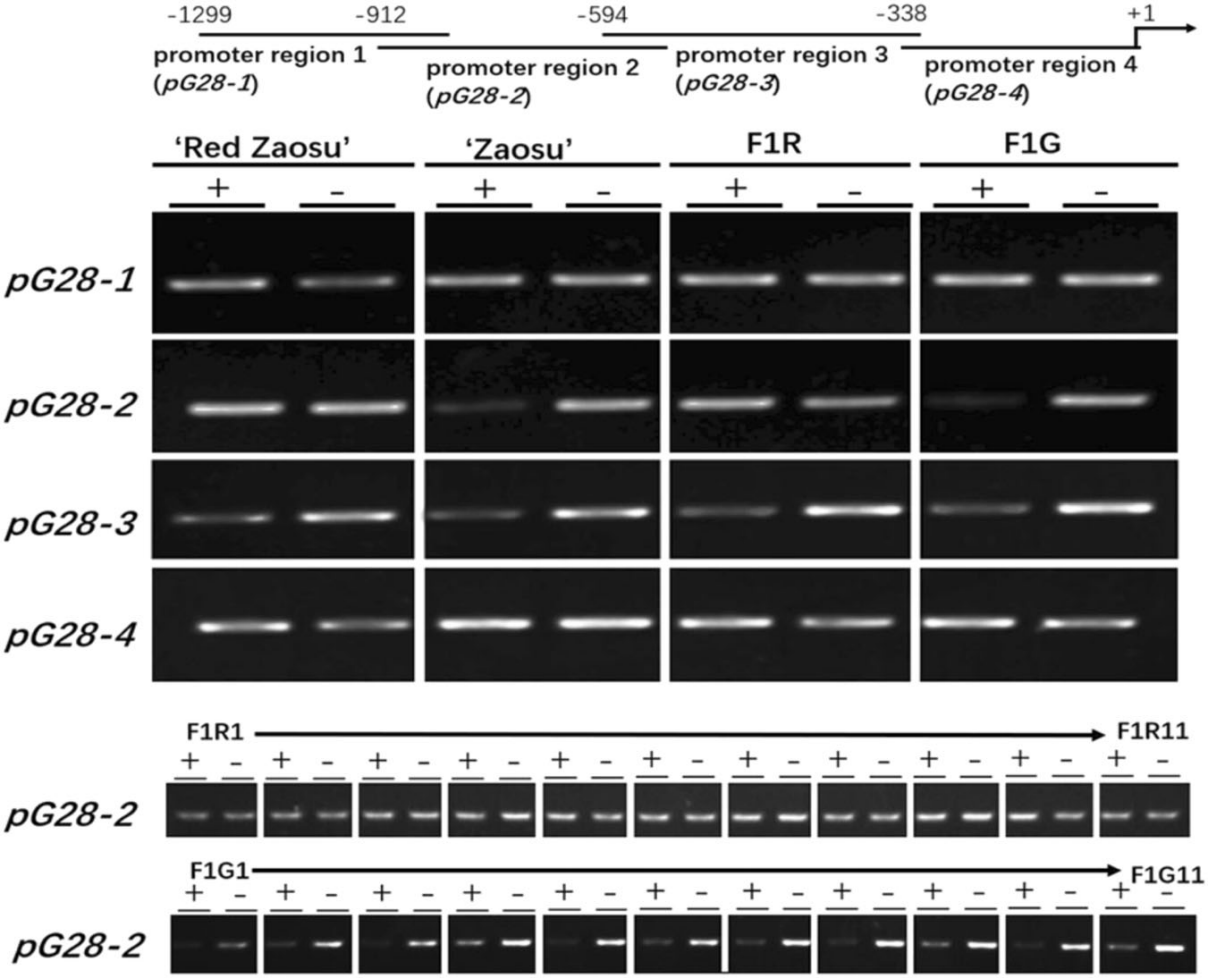
Methylation levels of the *PbGA2ox8* promoter regions among “Red Zaosu”, “Zaosu”, and their progeny as assessed by McrBc-PCR. The 1300-bp region upstream of the ATG translational start site of *PbGA2ox8* was divided into four overlapping fragments: promoter region 1 (*pG28-1*), promoter region 2 (*pG28-2*), promoter region 3 (*pG28-3*), and promoter region 4 (*pG28-4*). In total, 0.5 μg of genomic DNA was digested with McrBc in the presence GTP (+). Samples lacking GTP served as the negative controls (−). F1R and F1G, pooled red-, and green-leafed segregants, respectively, from the 15F1 population; F1R1–11 and F1G1–11, 11 randomly selected red and green-leafed individuals, respectively, from the 15F1 population; +1, ATG translational start site.

To determine why *PbGA2ox8* was expressed at atypical high levels in “Red Zaosu”, the promoter region of *PbGA2ox8* (1300 bp from the transcription initiation site) was further isolated from “Red Zaosu” and “Zaosu” (Supplementary Dataset S3). However, no sequence variation was identified in the “Red Zaosu” allele of the *PbGA2ox8* locus. Nevertheless, decreased methylation of the promoter region of “Red Zaosu” compared with “Zaosu” was identified using McrBC-PCR (Fig. 4).

The 1300-bp promoter was further divided into four overlapping regions (Fig. 4). The methylation levels in the *pG28-4*, *pG28-3*, and *pG28-1* regions were the same among “Red Zaosu”, “Zaosu”, F1R, and FIG. However, the methylation level in the *pG28-2* regions was lower in “Red Zaosu” and F1R than in “Zaosu” and F1G (Fig. 4). Moreover, a decreased methylation level in the *pG28-2* region was also identified in each red-leafed progeny from the 15F1 population compared with the corresponding green-leafed progeny (Fig. 4). Thus, the *pG28-2* region was demethylated exclusively in “Red Zaosu” and its red-leafed progeny. This result was consistent with the atypical overexpression of *PbGA2ox8* observed in “Red Zaosu” and its red-leafed progeny.

### *PbGA2ox8* overexpression reduced the GA_4_ level and induced anthocyanin accumulation in pear fruit

**Fig. 5:**
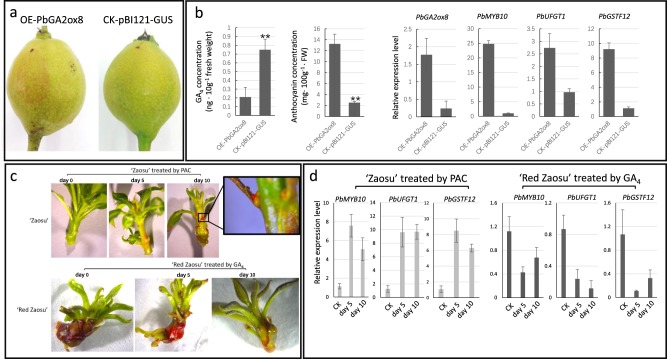
Anthocyanin accumulation associated with *PbGA2ox8* and GA_4_ in “Zaosu” and “Red Zaosu”. **a** Overexpression of *PbGA2ox8* (OE-PbGA2ox8) in “Zaosu” fruit. **b** GA_4_ concentration, anthocyanin concentration, and the expression patterns of *PbMYB10*, *PbUFGT1*, and *PbGSTF12* in OE-PbGA2ox8 fruit. **c** Effects of GA and PAC treatment on “Red Zaosu” and “Zaosu” seedlings. **d** The expression patterns of *PbMYB10*, *PbUFGT1*, and *PbGSTF12* under different treatment conditions. Error bars represent the means ± SEs of three biological replicates. Asterisks indicate significant differences (Student’s *t* test): **P < 0.01; *n* = 3.

To verify the function(s) of *PbGA2ox8* in pear, *PbGA2ox8* was transiently overexpressed in “Zaosu” fruit, resulting in a reduced GA_4_ concentration and increased anthocyanin biosynthesis (Fig. 5a, b). The typical anthocyanin regulatory transcription factor *PbMYB10* (homologous to *PAP1* in *Arabidopsis*), anthocyanin biosynthetic gene *PbUFGT1*, and anthocyanin transporter *PbGSTF12* were upregulated by *PbGA2ox8* overexpression (Fig. 5b).

Moreover, less bioactive GA_4_ was detected in the fruit skins and leaves of “Red Zaosu” than in those of “Zaosu”, but the GA_4_ levels in the receptacles of these two cultivars were similar (Supplementary Fig. S1). GA_4_ repressed anthocyanin accumulation in “Red Zaosu” subcultured explants within 10 days, and paclobutrazol (PAC), an inhibitor of bioactive GAs, induced anthocyanin accumulation in “Zaosu” within subcultured explants after 10 days (Fig. 5c). The expression levels of *PbMYB10*, *PbUFGT1*, and *PbGSTF12* were also upregulated by PAC in “Zaosu” and reduced by GA_4_ in “Red Zaosu” (Fig. 5d). Thus, *PbGA2ox8* regulated anthocyanin accumulation by adjusting the bioactive GA_4_ concentration in pear.

### The anthocyanin distribution corresponded to vascular bundles in “Red Zaosu”

After identifying the biofunction of *PbGA2ox8* in anthocyanin accumulation in “Red Zaosu”, we focused on the potential mechanism of stripe formation. Therefore, the anthocyanin distribution in “Red Zaosu” was investigated.

**Fig. 6:**
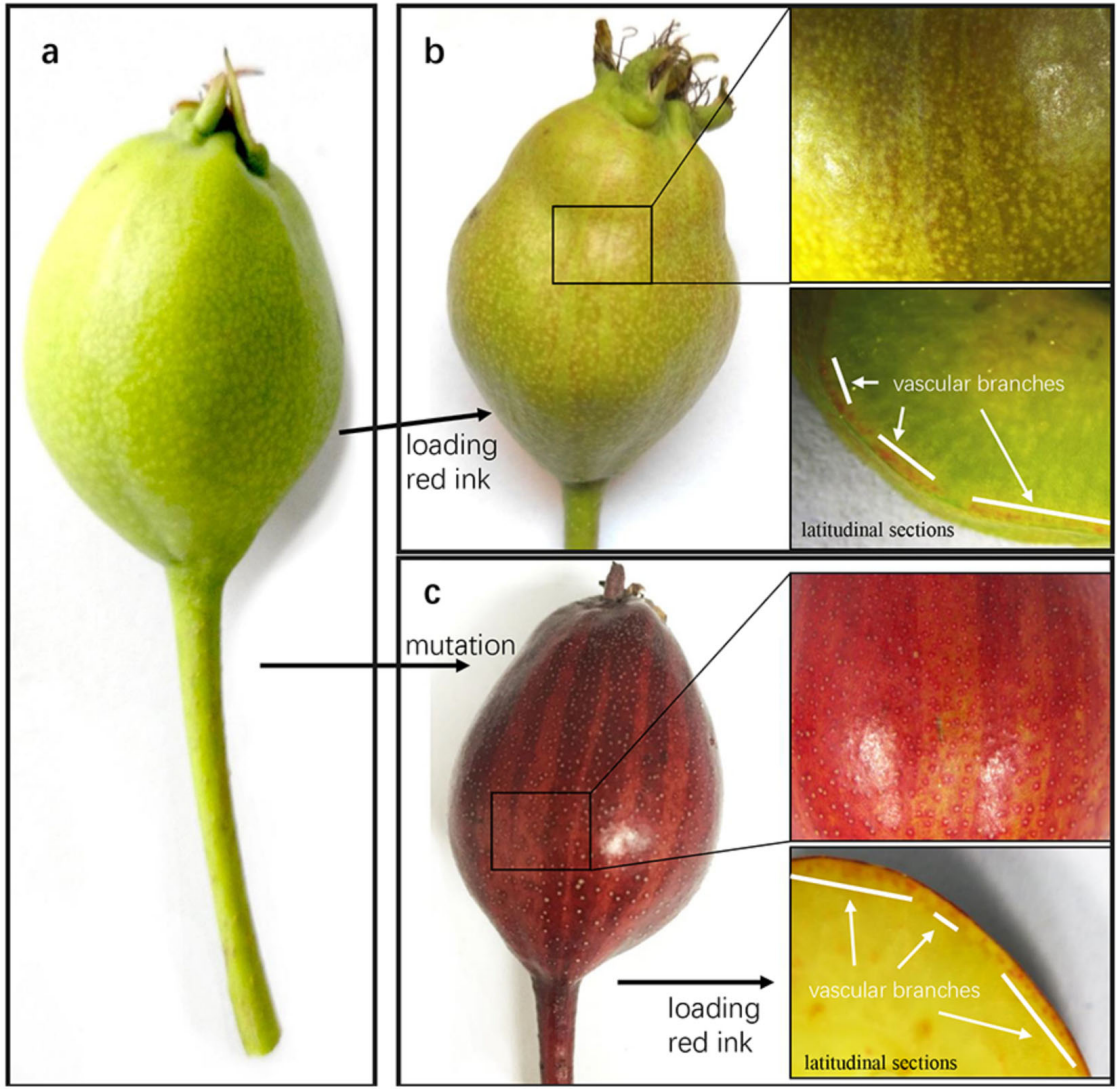
Vascular branches in the hypanthium and their spatial correlation with stripes on “Red Zaosu” fruit. **a** Unlabeled “Zaosu” fruitlet. **b** Violet ink-labeled “Zaosu” fruitlet. **c** Violet ink-labeled “Red Zaosu” fruitlet.

We initially identified vascular branches (VBs) near the fruit skin by loading them with violet ink or their observation in paraffinized sections (Fig. 6a–c; Supplementary Fig. S2). In wild-type “Zaosu”, the loading of violet ink in VBs produced a striped appearance on the hypanthial surface that was similar to that of “Red Zaosu” (Fig. 6b). In “Red Zaosu”, the violet ink-stained region was spatially coordinated with the red stripes in fruit (Fig. 6c). This result indicated that red stripe formation in “Red Zaosu” fruit is associated with the vascular system.

Moreover, large amounts of anthocyanin accumulated in epidermal cells in the leaf, petal, sepal, stamen, style, carpel, hypanthium, fruit stalk, and fruit skin of “Red Zaosu”. In contrast, limited anthocyanin production was observed in most tissues of “Zaosu“ (Supplementary Fig. S3a–n). The red stripes on “Red Zaosu” fruit were enriched in anthocyanins, and the stripes appeared on the “Red Zaosu” fruit surface immediately after receptacle formation were maintained during all of the fruit developmental stages, accompanied by red dots derived from the stomata (Supplementary Fig. S3d, h).

The red coloration of the leaf and petal of “Red Zaosu” was dendritic and occurred exactly along the veins derived from vascular bundles (Supplementary Fig. S3f, l). Tube-shaped organs, including the stamen and style, were also completely colored in “Red Zaosu” (Supplementary Fig. S3b, d). Major vascular bundles in the receptacles of “Zaosu” and “Red Zaosu” were classified as lateral carpellary, dorsal carpellary, and ventral carpellary bundles. The red regions in “Red Zaosu” were near dorsal carpellary bundles and ventral carpellary bundles (Supplementary Fig. S3b).

In summary, anthocyanins were enriched near vascular networks in the epidermal cells of fruit, leaves, and flower organs in “Red Zaosu”.

### Calcium was unevenly distributed and spatially coordinated with red stripes on pear fruit

**Fig. 7:**
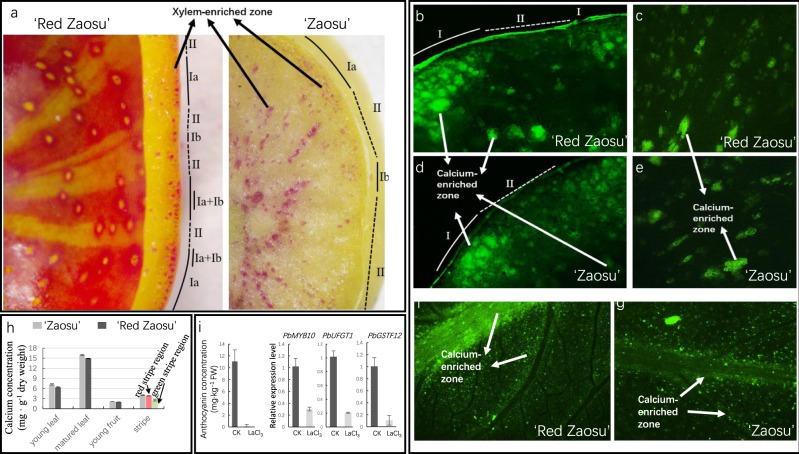
Calcium distribution in “Red Zaosu” and “Zaosu”. **a** Labeled xylem areas in “Red Zaosu” and “Zaosu”. **b**–**e** Fluorescence images of free calcium in the fruitlets of “Red Zaosu” and “Zaosu” labeled with Fluo-3/AM ester. Arrows indicate free calcium-enriched areas. **f**, **g** Fluorescence images of free calcium in the leaves of “Red Zaosu“ and “Zaosu” labeled with Fluo-3/AM ester. Arrows indicate free calcium-enriched areas. **h** Calcium concentrations in different tissues of “Red Zaosu” and “Zaosu”. **i** The anthocyanin concentration and related gene expression patterns in LaCl_3_-treated “Red Zaosu” fruit. Error bars represent the means ± SEs of three biological replicates.

The xylem cells, which are located mostly in the main vascular bundles among VBs and nearby lenticels (fruit dots) in fruitlets, were spatially coordinated with the red stripes and red fruit dots on “Red Zaosu” fruit surfaces (Fig. 7a). Based on their enrichment in xylem cells, two different areas near the fruit skin were classified. Type I zones in “Red Zaosu” fruit were red and enriched in xylem cells (type Ia was associated with the red stripe region; type Ib was associated with the red fruit dots). Type II zones in “Red Zaosu” fruit were green and lacked xylem cells (Fig. 7a).

Free calcium labeled with Fluo-3/AM ester was unevenly distributed and enriched in xylem cells in the fruit and leaves of “Zaosu” and “Red Zaosu” (Fig. 7b–g). Similar to the locations of xylem cells, the calcium distribution in the epidermal layers of fruit could be classified into two different regions in both pear cultivars. The type I zones were enriched in calcium (xylem), and type II zones lacked calcium (Fig. 7a–g). In summary, calcium-enriched areas in the epidermal layers of “Red Zaosu” fruit were spatially coordinated with red stripes on the fruit surface (Fig. 7).

Consistently, the total calcium concentrations quantified by atomic absorption spectrophotometry in “Red Zaosu” leaves were slightly lower than those in “Zaosu” (Fig. 7h). The calcium levels in the young fruit of “Red Zaosu” and “Zaosu” were similar, but calcium was enriched in red stripes but not green stripes in “Red Zaosu” (Fig. 7h).

Moreover, to identify whether calcium-mediated signaling participates in anthocyanin biosynthesis in “Red Zaosu” fruit, LaCl_3_ was applied to “Red Zaosu” fruit during its coloration. Hardly any anthocyanin accumulated in LaCl_3_-treated “Red Zaosu” fruit. The expression of anthocyanin-related genes was also blocked by LaCl_3_ (Fig. 7i).

### *PbGA2ox8* expression induced vascular-dependent anthocyanin accumulation

To investigate whether *PbGA2ox8*-induced anthocyanin biosynthesis is vasculature-related, *PbGA2ox8* was transiently overexpressed throughout the entire pear leaf by vacuum infiltration.

**Fig. 8:**
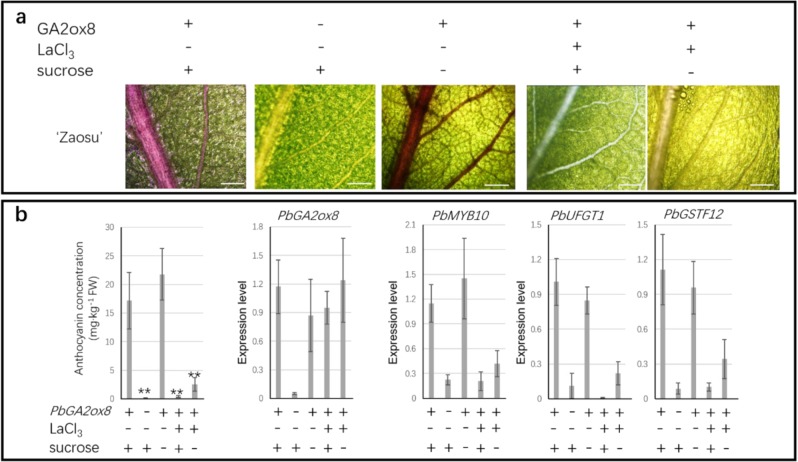
Effects of sucrose and calcium on *PbGA2ox8*-induced anthocyanin accumulation. **a** “Zaosu” leaves treated with different combinations of *GA2ox8* overexpression, sucrose and a calcium channel blocker (LaCl_3_). Scale bars, 1 mm. **b** The anthocyanin concentrations and expression patterns of *PbGA2ox8* and anthocyanin indicator genes. Error bars represent the means ± SEs of three biological replicates. Asterisks indicate significant differences (Student’s *t* test): **P < 0.01; *n* = 3.

The overexpression of *PbGA2ox8* in “Zaosu” induced vascular-dependent anthocyanin biosynthesis in the leaves of “Zaosu” (Fig. 8a). *PbMYB10*, *PbUFGT1*, and *PbGSTF12* were upregulated by *PbGA2ox8* overexpression (Fig. 8b). Because sucrose may regulate anthocyanin biosynthesis via the DELLA protein and can be transported by the vascular system^10^, the roles of both calcium and sucrose were investigated. *PbGA2ox8*-induced anthocyanin biosynthesis was not affected by the application of external sucrose to the leaves of “Zaosu” (Fig. 8a, b). However, calcium signal transduction was necessary for *PbGA2ox8*-induced anthocyanin biosynthesis in “Zaosu”. When calcium-mediated signal transduction was blocked by LaCl_3_, *PbGA2ox8*-induced anthocyanin biosynthesis was inhibited (Fig. 8a, b). In addition, *PbMYB10*, *PbUFGT1*, and *PbGSTF12* were not induced by *PbGA2ox8* in LaCl_3_*-*treated leaves (Fig. 8b). Thus, *PbGA2ox8* preferentially induced vascular-dependent anthocyanin biosynthesis in calcium-enriched areas.

## Discussion

Anthocyanin accumulation is responsible for the red coloration of some fruits, including apple, peach, strawberry, and pear. The key genes responsible for this trait have been studied. In apple, *MYB10* and *MYB110a* (homologous to PAP-type MYB transcription factors in *Arabidopsis*) have been mapped and identified as responsible for fruit color^31,32^. Other PAP-type MYB transcription factors, including *MYB10* in sweet cherry, *MYB10b* in pear, and *MYB10* in peach, have also been associated with anthocyanin accumulation in their respective fruit^19,33,34^. However, unlike these typical anthocyanin regulatory genes, in this study, we identified a novel gene that encodes *GA2ox8*, which contributes to the red fruit trait in the spontaneous bud sport “Red Zaosu”.

*PbGA2ox8* was expressed exclusively in the red organs of “Red Zaosu”, but not any other red pear cultivars tested in this study (Fig. 3). *GA2ox8* was found to hydroxylate GA_12_ and GA_53_ to inactive GAs^35^. A deficiency in bioactive GAs may induce anthocyanin accumulation by modulating DELLA proteins^12^. The red trait in “Red Zaosu” explants was reversed to green with GA_4_ treatment, and anthocyanin accumulation was observed in PAC (a GA biosynthetic inhibitor)-treated “Zaosu” explants (Fig. 5c, d). The overexpression of *AtGA2ox8* induced anthocyanin accumulation in *Brassica napus* L. leaves^36^. In this study, the function of *PbGA2ox8* was identified in transient assays. Such transient assays successfully demonstrated that the apple MYB transcription factor *MYB110a* regulated anthocyanin biosynthesis in leaves of *Nicotiana benthamiana* after infiltration. Anthocyanin accumulation induced by *MYB110a* was distributed evenly in the infiltrated area^32^. The overexpression of *PbGA2ox8* in young fruit by injection caused an anthocyanin-enriched cycle (Fig. 5). This result was consistent with previously reported GUS-labeled infection areas^19^. However, to investigate the anthocyanin distribution caused by *PbGA2ox8* overexpression, the whole leaf was infected by vacuum infiltration instead of injection. *PbGA2ox8* induced enriched anthocyanin accumulation near the vascular bundles in young leaves of “Zaosu” (Fig. 8).

To ascertain why *PbGA2ox8* was highly expressed in “Red Zaosu” but not any other red pear cultivars tested in this study, we cloned the promoter sequences of *PbGA2ox8* from “Red Zaosu” and “Zaosu”. However, no variations in sequence were identified (Supplementary Dataset S3). Instead, decreased methylation of the promoter region of *PbGA2ox8* was observed in “Red Zaosu” and its red-leafed progeny but not “Zaosu” and its green-leafed progeny (Fig. 4). In apple and pear, changes in the methylation of the MYB10 promoter were shown to regulate anthocyanin biosynthesis and affect fruit coloration^4,37^. A consistent correlation between the exclusively high expression level of *PbGA2ox8* and the decreased methylation of its promoter region in “Red Zaosu” and its red-leafed progeny was observed. Thus, the high level of *PbGA2ox8* expression observed in “Red Zaosu” might have resulted from the demethylation of its promoter region. Moreover, the cause of this epigenetic variation should be determined in a future study.

Comparative transcriptome analysis between “Red Zaosu” and “Zaosu” also indicated that anthocyanin accumulation in the former was associated with environmental stimuli and chemical signals (Fig. 2). Anthocyanin, which protects plants, can be induced by various stresses, including drought and osmotic pressure, as well as a deficiency in nitrogen or phosphorus^6,13,38^. Among the 26 DEGs screened, >50% responded to stress, and 27% were involved in signal transduction. Thus, anthocyanin accumulation in “Red Zaosu” might be a response to stress or chemical signaling (Fig. 2). However, the external environments of wild-type “Zaosu” and “Red Zaosu” were the same. Thus, internal signal transduction systems in the “Red Zaosu” sport might vary and be more sensitive than those in wild-type “Zaosu”, resulting in systemically different responses, including vascular-related anthocyanin biosynthesis.

Regular striping is easily observed on the fruit and flower stalks of some watermelon cultivars. This type of stripe formation may be associated with morphogen diffusion from vascular bundles^2^. Although stripes were also distributed on the flower stalks and sepals of “Red Zaosu”, the pattern of stripes on each “Red Zaosu” fruit was irregular (Supplementary Fig. S3h). The irregular red stripes observed on the hypanthial surface in “Red Zaosu” were further shown to coincide with the distribution of VBs in the hypanthium (Fig. 6). Similarly, models of stripe distribution in the other red organs of “Red Zaosu” showed that anthocyanins accumulated in the epidermal layers of leaf-derived tissues and corresponded to vascular networks.

After we identified the correlation between anthocyanin distribution and the vascular system, we determined that calcium was enriched in xylem cells near vascular bundles and in the stomata of both pear cultivars and spatially coordinated with the anthocyanin distribution in “Red Zaosu” (Fig. 7). Moreover, *PbGA2ox8*-induced anthocyanin biosynthesis was inhibited by LaCl_3_, indicating that calcium signaling participates in the anthocyanin biosynthesis process (Fig. 8). This result is consistent with the role of calcium signaling in the environmental regulation of anthocyanin biosynthesis^14,16^.

## Conclusion

**Fig. 9:**
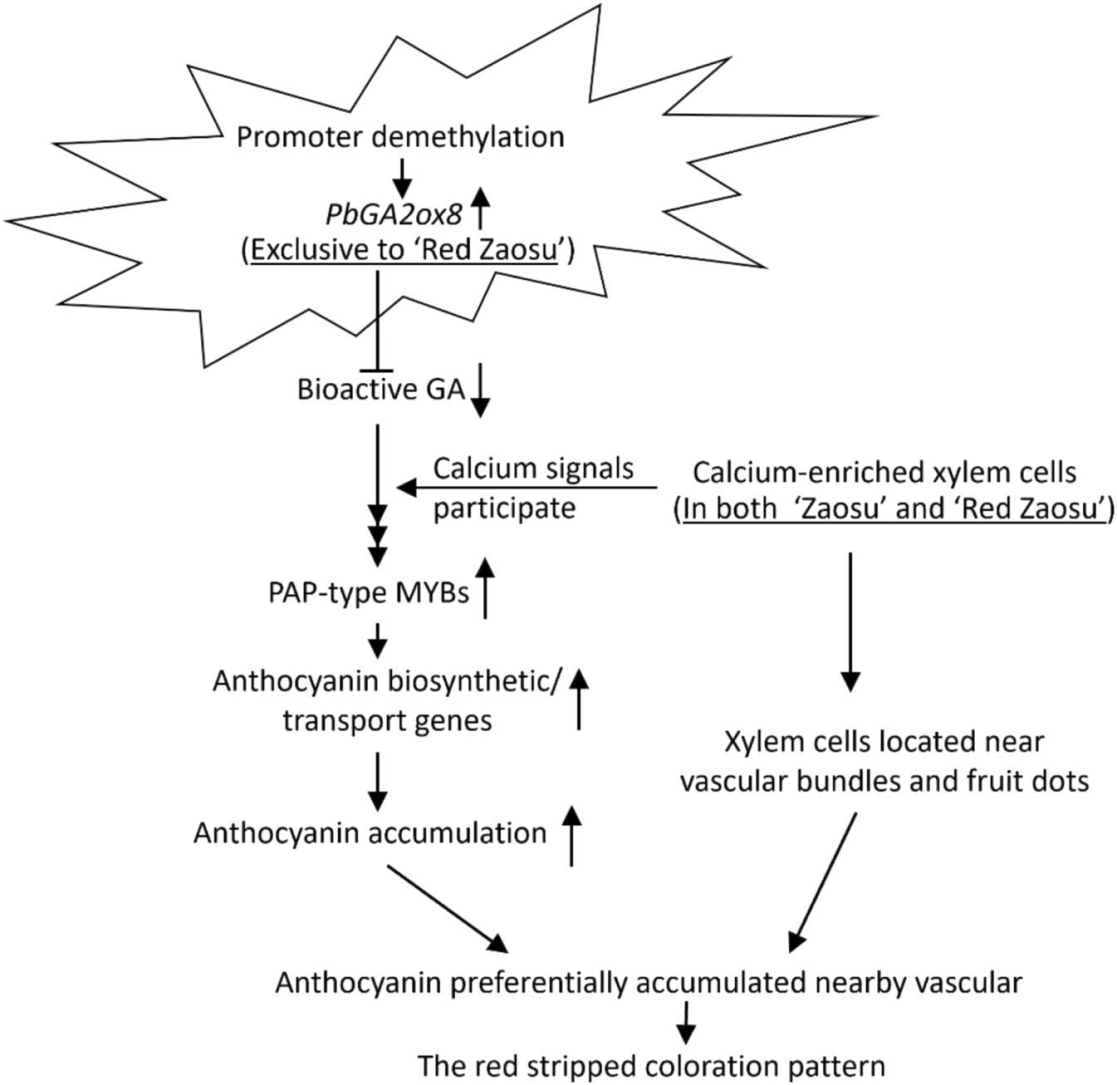
Mechanism of red stripe formation in the spontaneous pear mutant “Red Zaosu”.

We propose the following mechanism of anthocyanin stripe formation in this spontaneous pear sport. *PbGA2ox8* is expressed at a high level with a low level of promoter methylation exclusively in “Red Zaosu”, resulting in a low GA level. This activates the DELLA-mediated anthocyanin accumulation pathway. *GA2ox8*-induced anthocyanin accumulation occurs preferentially near longitudinal arrays of vascular bundles, resulting in permanent red stripes (Fig. 9).

## Supplementary information

revised Supplementary figures and tables

Dataset 3

Dataset 1

Dataset 2
