# Supplementary material for: PbGA2ox8 induces vascular-related anthocyanin accumulation and contributes to red stripe formation on pear fruit
Source: Hortic Res. 2019 Dec 1;6:137. doi: 10.1038/s41438-019-0220-9 (PMC6885050; doi:10.1038/s41438-019-0220-9)
Supplement: Supplementary file 1 — revised Supplementary figures and tables [file 41438_2019_220_MOESM1_ESM.docx]

**Figure S1.** GA_4_ concentrations in different tissues of ‘Red Zaosu’ and ‘Zaosu’. Error bars represent ±SE of three biological replicates


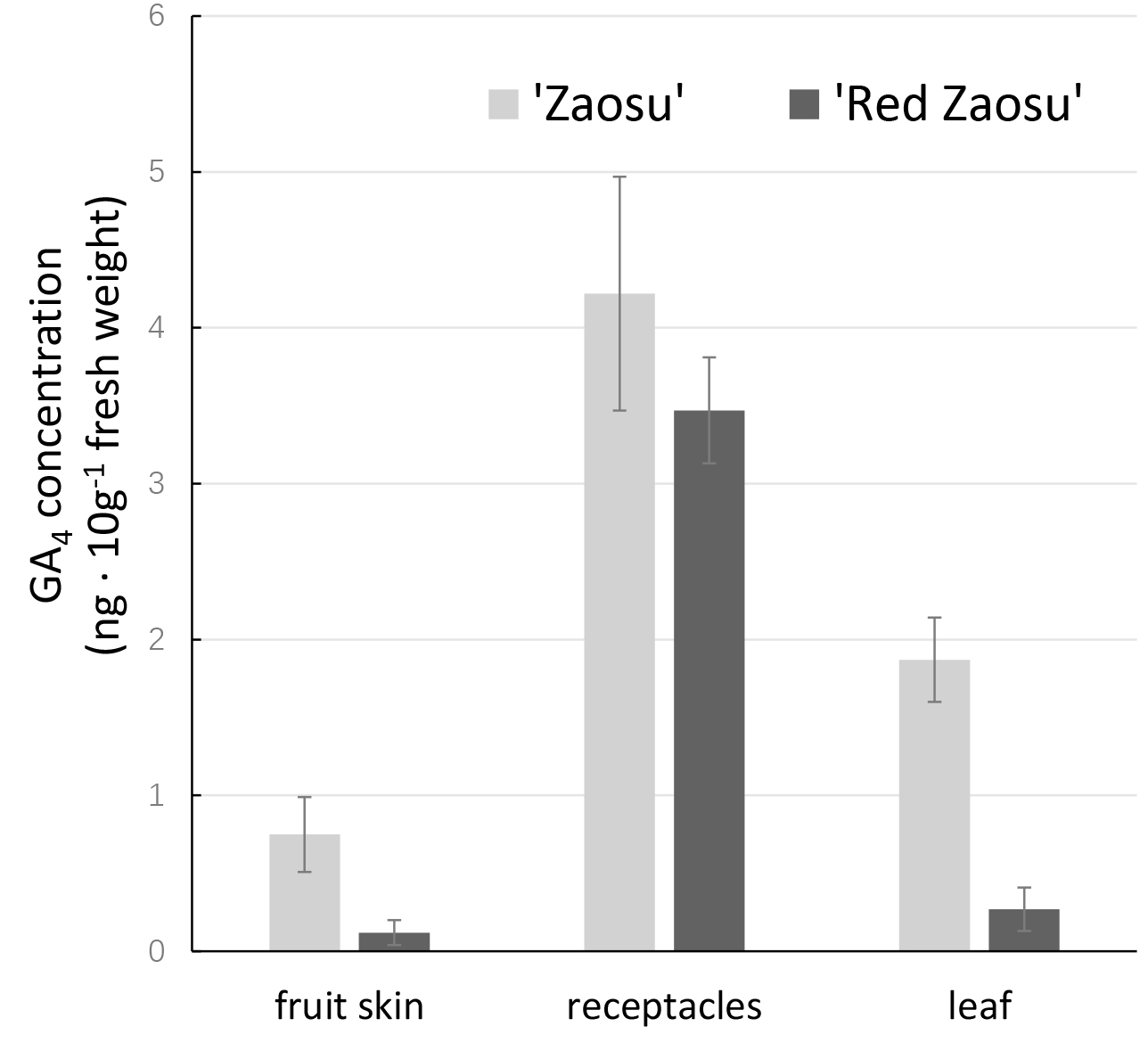


**Figure S2**. Fluorescence-labelled vascular branches in paraffin sections of ‘Red Zaosu’ receptacle**
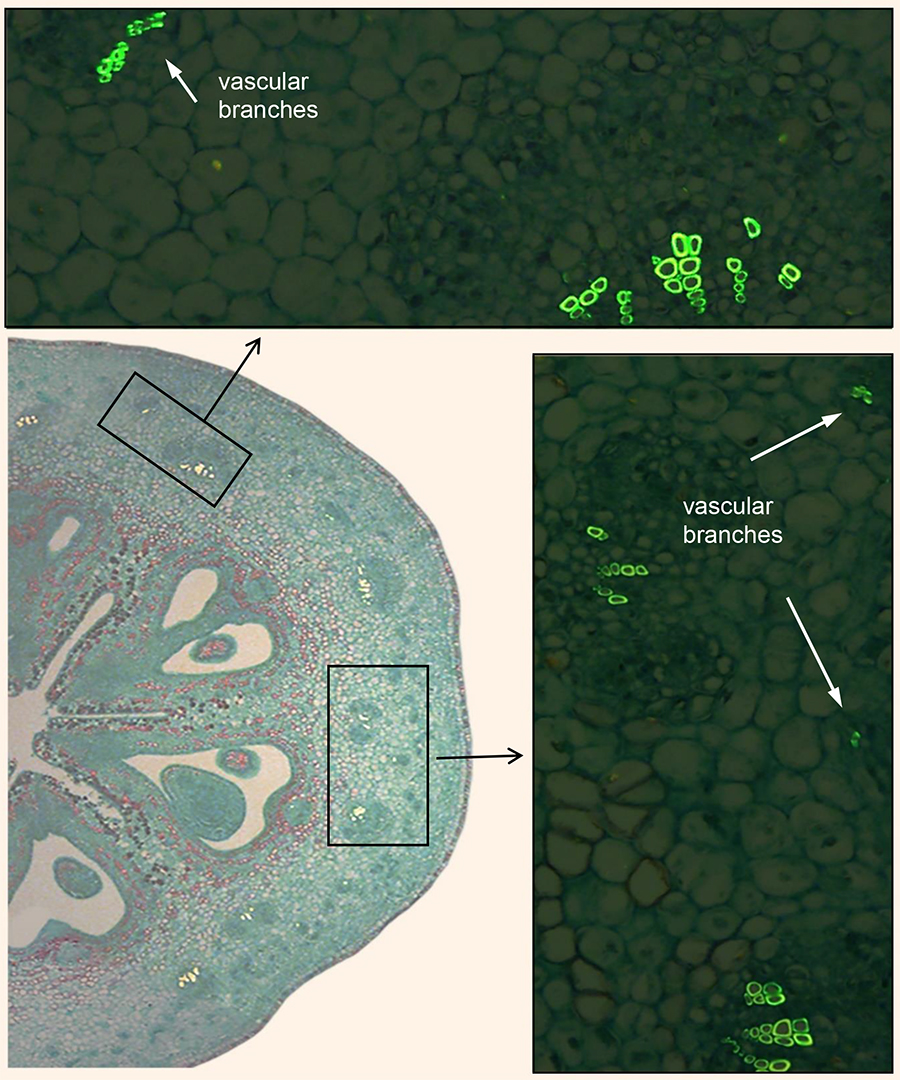
**

**Figure S3. The appearance of different tissues and the anthocyanin distribution pattern in the ‘Red Zaosu’ mutant and the wild type ‘Zaosu’ pear.** Scale bars, 1.5 cm (g to l), 1 mm (a to f, e). (a) and (b) Latitudinal sections of receptacles of ‘Red Zaosu’ and ‘Zaosu’, respectively. (c) and (d) Receptacles of ‘Red Zaosu’ and ‘Zaosu’, respectively. (e) and (f) Sepals of ‘Red Zaosu’ and ‘Zaosu’, respectively. (g) and (h) Mature fruit of ‘Red Zaosu’ and ‘Zaosu’, respectively. (i) and (j) Latitudinal sections of mature fruit of ‘Red Zaosu’ and ‘Zaosu’, respectively. (k) and (l) Mature leaves of ‘Red Zaosu’ and ‘Zaosu’, respectively. (m) fruit stalk and receptacles of ‘Red Zaosu’. (n) anthocyanin concentrations among different tissues of ‘Red Zaosu’ and ‘Zaosu’. a, c, e, g, i and k represent different tissues of ‘Zaosu’. b, d, f, h, j, l and m represent different tissues of ‘Red Zaosu’. Error bars represent SE. Asterisks indicate significant differences (Student’s ttest): **, P< 0.01; n= 3.

**
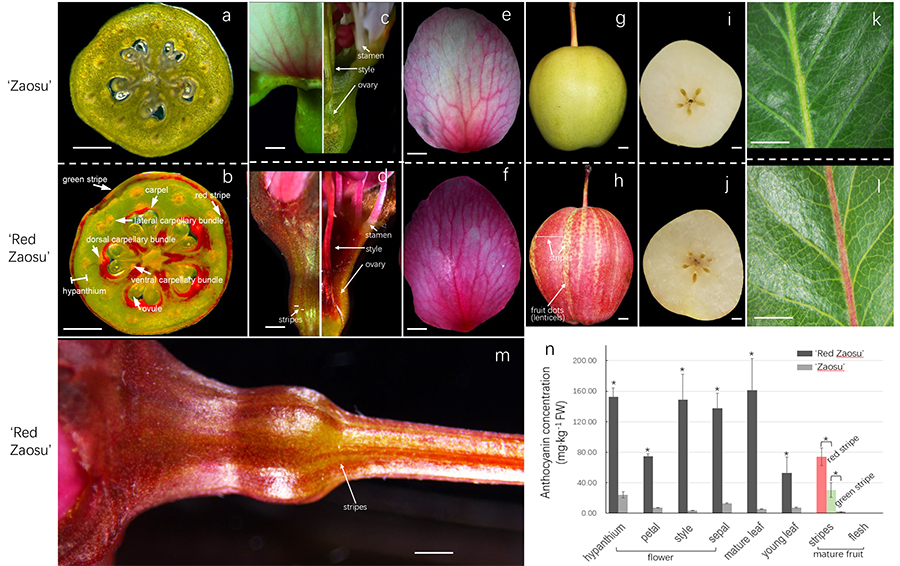
**

**Table S1** Detailed information of plant materials

| Cultivars | Tissue type | Cultural condition | | Sampling date |  |
| --- | --- | --- | --- | --- | --- |
| 'Red Zaosu' | flower (receptacle, hypanthium, petal, style, sepal) | natural growth | | 2 days before full bloom (April 3rd, 2017)/1 day after full bloom (April 6th, 2017) |  |
|  | young leaf | natural growth | | 60 days after full bloom (June 4th, 2017) |  |
|  | carpopodium | artifical bagging for 30 ± 5 days | | 45 days after full bloom (May 19th, 2017) |  |
|  | mature leaf | natural growth | | 60 days after full bloom (June 4th, 2017) |  |
|  | mature fruit | natural growth /artificial bagging for 90 ± 5 days days | | 105 days after full bloom (July 19th, 2017) |  |
|  | bagged fruit | immersed in 5 mM LaCl_3_ for 12 hours and then exposed to sunlight | | 5 days after exposure to sunlight |  |
|  | explants | cultured in MS medium at 24℃ with 16 hours photoperiod at 3600lx light intensity (artificial shading at 1200lx) | | 30 ± 5 days after subculture |  |
|  | explants | subcultured in MS medium contaning 7.5 μM GA_4_ | | 5 days and 10 days after subculstued |  |
|  |  |  | |  |  |
| 'Zaosu' | flower (receptacle, hypanthium, petal, style, sepal) | natural growth | | 2 days before full bloom (April 3rd in 2017)/1 day after full bloom (April 6th in 2017) |  |
|  | young leaf | natural growth | 60 days after full bloom (June 4th, 2017) | | |
|  | carpopodium | natural growth | 45 days after full bloom (May 19th, 2017) | | |
|  | mature leaf | natural growth | 60 days after full bloom (June 4th, 2017) | | |
|  | mature fruit | natural growth | 105 days after full bloom (July 19th, 2017) | | |
|  | explants | cultured in MS medium at 24℃ with 16 hours photoperiod at 3600lx light  intensity (artificial shading cultured at 1200lx) | 30 ± 5 days after subculture | | |
|  | explants | subcultured in MS medium contaning 5 μM PAC | | 5 days and 10 days after subculstued |  |
|  |  |  | |  |  |
| 'Red Anjou' | young leaf | natural growth | | 60 days after full bloom (June 4th, 2017) |  |
| 'Hong sichou' | young leaf | natural growth | | 60 days after full bloom (June 4th, 2017) |  |
| 'Early red Comice’ | young leaf | natural growth | | 60 days after full bloom (June 4th, 2017) |  |
| 'Dang shansu' | young leaf | natural growth | | 60 days after full bloom (June 4th, 2017) |  |
| 'Barlett' | young leaf | natural growth | | 60 days after full bloom (June 4th, 2017) |  |
| 'Suisho' | young leaf | natural growth | | 60 days after full bloom (June 4th, 2017) |  |
|  |  |  | |  |  |
| F1 seedlings of 'Red Zaosu' ×'Yuluxiang' | young leaf | natural growth | | June 25th, 2017 |  |
| *pear cultivars were 10 years old and drafted on *Pyrus betulaefolia* Bunge | | | | |  |

**Table S2** Primers and gene accessions in this study

|  | Accession | Gene name | Forward primer (5'-3') | Reverse primer (5'-3') |
| --- | --- | --- | --- | --- |
| qPCR | LOC103927277 | *MYB110a* | GCACGTCACTGGCCCTTATT | ACTAGCCACTTCAACGCCTC |
|  | LOC103929477 | *PLT1* | GCCAATGCAATGCTCAAGGA | ACATATGTCACAACAAAAACCA |
|  | LOC103933269 | *CHLH* | CCCGAAGTGGTACGAAGGAA | GCCTCTTCGTACACCCAGTT |
|  | LOC103952407 | *PLA-I1* | TTCCGGCCGTGTGTTTAGAA | AGCCCGGATTAGGGAAAACC |
|  | LOC103952446 | *PbMYB10b* | AGAACAAAAGCCACATTCGTC | GTAGGTGGTGATGATGTCCGTA |
|  | LOC103932710 | *BAP2* | CGGTTTGGTACGGTGCCTAT | GCATCAAATCTCCTGCAAGAAC |
|  | LOC103946101 | *ECR* | GTGCTAGGGTTTCCAGAGCC | TCTCTTCACCTGGCTTCGAC |
|  | LOC103933798 | *UGT85A23* | CCCACCACCACACCATTTCTT | GACCTTGAGCTGGGAATGGTA |
|  | LOC103926530 | *XND1* | TGGATAATGCATGAGTATCGTCTAT | CTGTATTCTAACTGTTTTGGGTTGC |
|  | LOC103948768 | *GA2OX8* | TCGTACTGTCCTGGATTCCT | TGCCTTCGAATCTGTACCCG |
|  | LOC103951140 | *ADH1* | AGGGGAAGGTCTTCGTTGC | ACAAAGAAAACCGAAAGACGAA |
|  | LOC103953275 | *PCAP1* | GGCCAGCCTATGTTTCAGGT | TTTGTTGCTTCTGCTGGCG |
|  | LOC103966123 | *DTX35* | GGCAAGAGTAAGAAGGCGGT | GGATGGGGAGTGCACTGTAG |
|  | LOC103959467 | *PbMYB10* | TGAAGTGGGTAGCAGGCAAA | TCGAGTCCAGGCACCTTTTC |
|  | LOC103959931 | *PbGSTF12* | TTGAGGCAGGAGAGCACAAG | CCTGCATACTTGGCTGCGTA |
|  | LOC103937481 | *PbUFGT1* | CTGGAACCTGAAGTTGTGAATCTG | AGCCACTCTAAGCAACCACTATC |
|  | LOC103926850 | *PbActin* | CCATCCAGGCTGTTCTCTC | GCAAGGTCCAGACGAAGG |
|  |  |  |  |  |
| CDS | LOC103948768 | *PbGA2OX8* | ATGGATTTCAAACCTCCCTTC | TCAAAGGAGAAATCTTGAGAGC |
| Infusion | LOC103948768 | *PbGA2OX8* | GGACTCTTGACCATGGATGGATTTCAAACCTCCCTTCCAACA | ATTCGAGCTGGTCACCTCAAAGGAGAAATCTTGAGAGCCCTACT |
